# Using Post-Mortem Measurements to Predict Carcass Tissue Composition in Growing Rabbits

**DOI:** 10.3390/ani12050605

**Published:** 2022-02-28

**Authors:** Andrea Y. Croda-Andrade, Cristell G. Valencia-García, Tomas A. Arbez-Abnal, Rodrigo Portillo-Salgado, Raciel J. Estrada-León, Ignacio Vázquez-Martínez, Enrique Camacho-Pérez, Einar Vargas-Bello-Pérez, Alfonso J. Chay-Canul

**Affiliations:** 1 División Académica de Ciencias Agropecuarias, Universidad Juárez Autónoma de Tabasco, Villahermosa 86280, Mexico; croda668@hotmail.com (A.Y.C.-A.); cripyvg98@gmail.com (C.G.V.-G.); rps_1303@hotmail.com (R.P.-S.); 2 Tecnológico Nacional de México, Instituto Tecnológico de Conkal, Conkal 97345, Mexico; tomas.arbaez@itconkal.edu.mx; 3 Tecnológico Nacional de México, C.A. Bioprocesos, Instituto Tecnológico Superior de Calkini, Calkini 24900, Mexico; rjestrada@itescam.edu.mx; 4 Programa de Ingeniería Agroforestal, Complejo Regional Norte, Benemérita Universidad Autónoma de Puebla, Tetela de Ocampo 73640, Mexico; ignacio.vazquez@correo.buap.mx; 5 Instituto Tecnológico Nacional de México, Instituto Tecnológico Superior Progreso, Progreso 97320, Mexico; enrique.camacho@gmail.com; 6 Department of Veterinary and Animal Sciences, Faculty of Health and Medical Sciences, University of Copenhagen, DK-1870 Frederiksberg C, Denmark

**Keywords:** carcass, growing rabbits, body muscle, prediction

## Abstract

**Simple Summary:**

The objective of this study was to determine postmortem measurements for predicting carcass traits in growing rabbits. A total of 50 New Zealand White × Californian male rabbits were used. Data recorded at slaughtering included carcass and noncarcass components (viscera and offal). Our results indicated that the use of carcass measurements could accurately and precisely (r = ≥0.76 and ≤0.84) be used as alternatives to predict the carcass weights and carcass tissues in growing rabbits.

**Abstract:**

The objective of this study was to determine post-mortem measurements for predicting carcass traits in growing rabbits. A total of 50 clinically healthy New Zealand White × Californian male rabbits with a body weight (BW) of 1351 ± 347 g between 60 to 80 days of age were used. Body weight was recorded 12 h before slaughtering. Data recorded at slaughtering included carcass weights (HCW). After cooling at 4 °C for 24 h, carcasses were weighed (CCW) and then were carefully split longitudinally with a band saw to obtain left and right halves. In the right half carcass, the following measurements were recorded using a tape measure: dorsal length (DL), thoracic depth (TD), thigh length (TL), carcass length (CL), lumbar circumference (LC). The compactness index (CCI) was calculated as the CCW divided by the CL. Thereafter, the right half carcass was weighed and manually deboned to record weights of muscle (TCM), and bone (TCB). The CCI explained of 93% of variation for TCM (R^2^ = 0.93 and a CV = 9.30%). In addition, the DL was the best predictor (*p* < 0.001) for TCB (R^2^ = 0.60 and a CV = 18.9%). Our results indicated that the use of carcass measurements could accurately and precisely (R^2^ = ≥ 0.60 and ≤0.95) be used as alternatives to predict the carcass tissues composition in growing rabbits.

## 1. Introduction

In Mexico, per capita consumption of rabbit meat varies between 30 and 134 g per person per year, and in recent years there has been a growing interest in the production of rabbits [1,2]. Rabbit production is a favourable activity for small and medium producers, due to the versatility of this species, the little investment required, and the possibility of generating income throughout the year [3]. However, rabbit production systems in Mexico have been hampered by certain factors, such as the lower economic importance of this species compared to livestock and poultry production and limited information on their management, reproduction, and productivity, including carcass yield and carcass characteristics [4].

In Mexico, the main rabbit breeds for meat production are New Zealand and California [5]. From an economic point of view, in rabbit production, carcass yield is the most important trait [6]. The evaluation of the carcass allows them to be classified according to carcass weight and carcass tissue composition and distribution [5]. Therefore, in rabbit production, knowing carcass traits should be feasible and practical to assess [7]. This information could help to improve genetics more quickly without the need for lengthy progeny testing to determine the merit of carcass traits [8].

In this sense, a professional’s measurements could be a means of describing body size and conformation, which are important traits in meat animals. On the other hand, it is important to know the relationship between certain carcass traits and carcass measurements in rabbits [7,9]. Knowledge of the carcass tissue composition and its distribution in rabbits is valuable information because one of the main challenges in the market of meat, along with ensuring food safety, is the commercialization of meat and meat products. For that, is necessary to generate information on carcass tissue composition to provide valuable information to improve the economic viability of rabbit production systems [2]. Until now, information on the use of post mortem measurements for predicting carcass traits of growing rabbits raised in tropical conditions is scarce. Therefore, the objective of the present study was to determine post mortem measurements for predicting carcass traits in growing rabbits.

## 2. Materials and Methods

### 2.1. Experimental Site and Animals

All animals were managed in compliance with the guidelines and regulations for ethical animal experimentation of the División Académica de Ciencias Agropecuarias, Universidad Juárez Autónoma de Tabasco (ID project PFI: UJAT-DACA-2015-IA-02). The climate (Am) of the region is tropical humid with rains in the summer, altitude is 9 m above sea level, with an average annual rainfall of 1958 mm, a relative humidity close to 75%, and an average annual temperature of 27 °C.

In this study, 50 clinically healthy New Zealand White × Californian fattening male rabbits with body weight (BW) of 1351 ± 347 g and between 60 to 80 days of age were used. All animals were obtained from a commercial farm and were fed a standard commercial diet (17% crude protein, 11% crude fibre, 2% fat, and 11% ash). Feed and water were provided ad libitum. Rabbits were housed in individual raised-slatted floor cages (45 × 30 × 40 cm), having a photoperiod of 10 h and natural ventilation.

### 2.2. Slaughter of Animals and Carcass Measurements

Feed and water were withdrawn 12 h before slaughtering, and BW was recorded. Animals were slaughtered according to the Mexican Official Standard NOM-033-SAG/ZOO-2014 for the humane slaughtering of animals. After slaughtering, hot carcass weight (HCW) was recorded. After cooling at 4 °C for 24 h, carcasses were again weighed (CCW) and then were carefully split longitudinally with a band saw to obtain left and right halves. In the right half carcass, the following measurements were recorded: dorsal length (DL) was considered as the interval between the first cervical vertebra and the seventh lumbar vertebra; thoracic depth (TD) was considered between the fifth and seventh thoracic vertebra and longitudinally surrounding the ribs until ending at the sternum; the thigh length (TL) was the interval between the seventh lumbar vertebra and the distal part of the ischium; the carcass length (CL) was calculated as the sum of the dorsal length and thigh length data; the lumbar circumference (LC) was the circumference of the carcass at the level of the seventh lumbar vertebra. Measurements were performed using a tape measure [10]. With this information, the compactness index (CCI) was calculated as the CCW divided by the carcass length. Thereafter, the right half carcass was weighed and manually deboned to record weights of muscle (TCM), and bone (TCB) [11]. Dissected tissues of the right half carcass were adjusted as whole carcasses. Carcass management was carried out at the Meat and Meat Products Technology Laboratory from Universidad Juárez Autónoma de Tabasco.

### 2.3. Data Analyses

For the statistical analysis and internal validation of the model, the data were read in the Python environment as follows: descriptive statistics were obtained using the description function of the “pandas” package [12]. The relationship between carcass traits and carcass measurements was determined by linear regression equations using the “lmfit” package [13]. The models and their residuals were plotted with the “matplotlib” package [14]. The goodness-of-fit of the regression models was evaluated using the Akaike Information Criterion (AIC), the Bayesian Information Criterion (BIC), the coefficient of determination (R^2^), the mean square error (MSE), and the root of MSE (RMSE). The last three parameters were obtained using the “scikit-learn” package [15]. The predictive capacity of the three models was evaluated by cross-validating *k*-folds (*k* = 10). This approach was undertaken by randomly dividing the set of observation values into nonoverlapping *k*-folds of approximately the same size. The first fold is treated as a validation set, and the model fits the remaining *k* − 1 folds (training data). The ability of the fitted model to predict the actual observed value was evaluated using the mean square error of prediction (MSEP), the root mean square error of prediction (RMSEP), and the R^2^ mean absolute error (MAE). The MSEP was calculated as the squared distance between the predicted value and the true value. The RMSEP was calculated by summing all squared prediction errors during cross-validation and is an indicator of the reliability and predictive ability of the model. The lower the RMSEP value, the higher its predictive ability for the model.

The MAE was calculated by taking the summation of the absolute difference between the actual and calculated values of each observation over the entire array and then dividing the sum obtained by the number of observations in the array. Lower values of root RMSEP and MAE indicate a better fit. The *k*-folds cross-validation was performed using the “scikit-learn” package [15], which allowed a comparison of numerous multivariate calibration models.

## 3. Results

The descriptive statistics of body weight, carcass traits, and postmortem measurements are presented in Table 1. Body weight ranged from 718 to 2491 g, while HCW and CCW ranged from 297 to 1390 g and 280 to 1334 g, respectively. With regard to postmortem measurements, mean values of DL, TD, CL, TL, and LC were 26.55 ± 2.83, 9.71 ± 1.38, 31.78 ± 3.18, 5.27 ± 0.97, and 13.98 ± 2.02 cm, respectively. The mean value for ICC was 21.63 ± 5.15 g/cm.

The correlation coefficients (r) between body weight, carcass traits and post mortem measurements are presented in Table 2. Except relationship between TCB and TL (*p* > 0.05), significant positive correlations were obtained between body weight and carcass traits with all post mortem measurements. The correlation coefficient (r) between the variables ranged from moderate to high (r = 0.47 to 0.97). The BW was highly correlated with CCI (r = 0.96), LC (r = 0.87), and TD (r = 0.86). On the other hand, both HCW and CCW had high correlations with CCI (r = 0.97 and r = 0.97, respectively) and LC (r = 0.91 and r = 0.90, respectively). Finally, TCM strongly correlated with CCI (r = 0.96) and LC (r = 0.90), while TCB showed moderate correlations with DL (r = 0.77) and CL (r = 0.75).

Significant predictors, determination coefficient (R^2^), mean square error (MSE) and *p*-value for predicting the carcass tissue composition are shown in Table 3. For the prediction of TCM, the CCI was again the most significant variable (R^2^ = 0.93 and CV = 9.30%, *p* < 0.001), but the precision showed a slight increase at R^2^ = 0.95 when the CL was added to the prediction model. On the other hand, the most significant predictor variable for total TCB was DL with R^2^ = 0.60 and CV = 18.9%.

**Figure 1:**
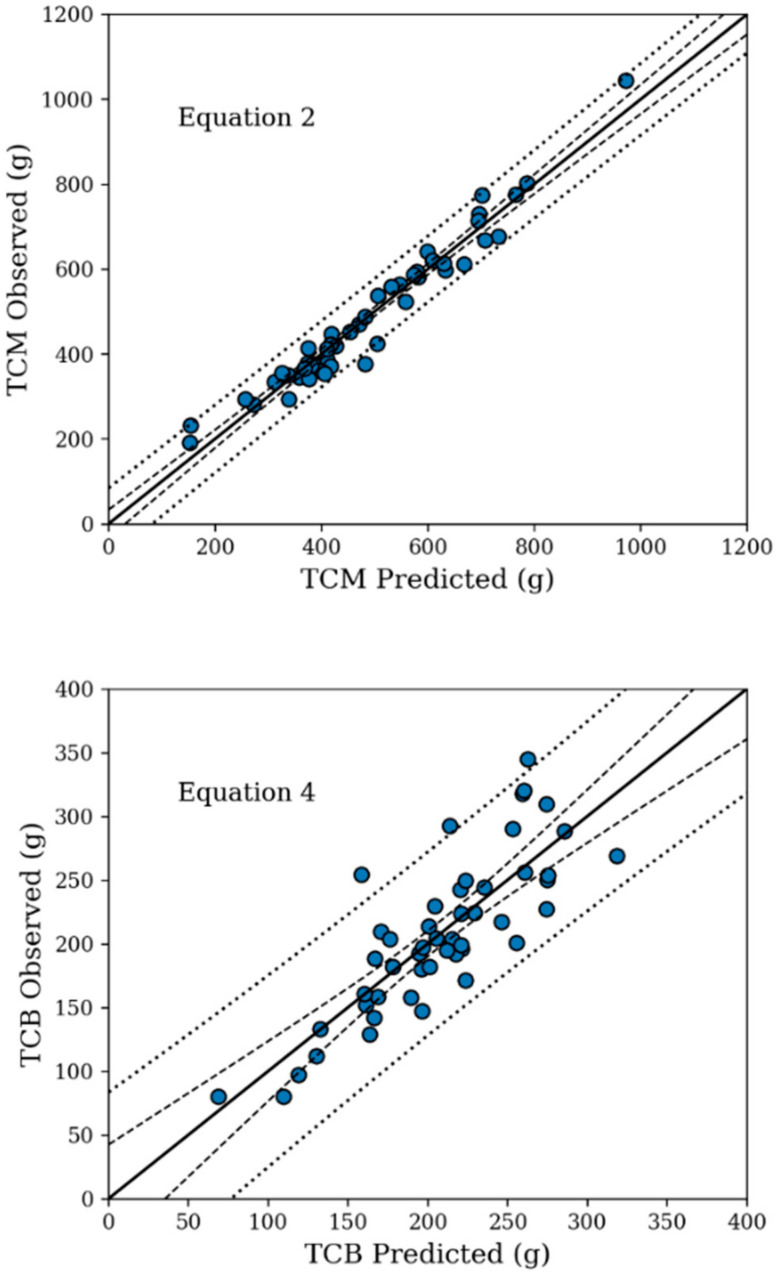
Relationship between observed and predicted values of carcass traits in growing rabbits, the best equations are presented.

The goodness-of-fit of the equations was calculated by the *k*-folds cross-validation technique is shown in Table 4. The proposed models showed adequate goodness-of-fit based on internal validation. The equations showed good performance according to the goodness-of-fit evaluation and internal validation (Table 4). With exception of the model for predicting TCB, all models had an R^2^ ≥ 0.93; however, the values of RMPE indicated a good performance of the fitted model for predicting carcass tissue composition. These models may be used both in experimental and commercial farms to predict carcass traits in growing rabbits (Figure 1).

## 4. Discussion

In general, mean values and ranges of variation for HCW and CCW were lower than those reported by Ortiz-Hernández and Rubio-Lozano [16] in different rabbit breeds. Such differences are due to the different mean values of rabbits’ body weights used in that study (~2000 g) versus this study (1329 g). Paci et al. [17] have previously reported that slaughter weight, age, and genotype are important factors that affect variability in the performance and composition of rabbit carcass. The observed carcass yield was consistent with that reported by Montes-Vergara et al. [6] in New Zealand White (NZ)-breed rabbits, however, in the present study animals were New Zealand White × Californian rabbits.

In the study, the highest correlation was observed between hot carcass weight and cold carcass weight with the carcass compactness index. Previously, Venturini et al. [18] showed in lambs that the higher the cold carcass weight, the higher the observed carcass compactness index with a correlation coefficient of r = 0.95, consistent with the results of this study. The carcass compactness index was highly correlated with the total carcass muscle, while the dorsal length correlated with the total carcass bone. These relationships could be because the carcass compactness index is a strong indicator of carcass conformation, as it evaluates the amount of muscle tissue deposited in the carcass in a unit of length. This is important in economic terms since the market for meat has a preference for more compact carcasses and more muscle tissue [19]. Michalik et al. [7] reported in French Lop rabbits that the total meat weight was correlated with hips circumference (r = 0.69), thigh circumference (r = 0.68), and pelvis width (r = 0.66), while the total bone weight was correlated with the pelvis width (r = 0.73) and thigh length (r = 0.65).

The high coefficients of determination of the regression equations obtained in the present study indicated that carcass compactness index could be used as the only variable to accurately predict body weight and the carcass traits. However, the precision could increase when considering the carcass length in the prediction models. Earlier, Blasco et al. [20] reported that total carcass muscle in California rabbits could be correctly predicted using only carcass weight or slaughter weight as a predictive variable, showing correlation coefficients of r = 0.88 and r = 0.84, respectively; therefore, it was not essential to add more predictor variables to the models. Later, Hernández et al. [10] showed that the composition of the carcass in rabbits can be clearly defined using the weight of the carcass, the meat/bone ratio of the hind leg, and the weight of the perirenal fat deposit. Recently, Michalik et al. [7] reported that the meat weight in the whole carcass in French Lop rabbits can be predicted (r = 98) using pelvic width, chest girth, thigh circumference, and carcass weight. These equations can be applied in selection works aimed at improving the meatiness of the carcass of the species.

The cross-validation of equations obtained in the present study, indicated a good performance according to the goodness-of-fit evaluation based on cross-validation because the values of RMSEP indicated a good performance of the fitted model when predicting carcass traits.

## 5. Conclusions

Our results indicated that the use of carcass measurements could accurately and precisely (R^2^ = ≥0.60 and ≤0.95) be used as alternatives to predict carcass weights and carcass tissues from growing rabbits. The equations showed good performance according to the goodness-of-fit evaluation based on cross-validation. With exception of the model for predicting TCB, all models had an R^2^ ≥ 0.93, in addition to the values of RMSEP indicating the good performance of the fitted model in predicting the carcass tissue composition.

## Figures and Tables

**Table 1:** Descriptive statistics of body weight (g), carcass traits (g), and postmortem measurements (cm) in growing rabbits (*n* = 50).

Variable	Description	Mean ± SD	Maximum	Minimum
BW	Body weight (g)	1351 ± 347	2491	718
HCW	Hot carcass weight (g)	698 ± 221	1390	297
CCW	Cold carcass weight (g)	696 ± 171	1334	280
DL	Dorsal length (cm)	26.5 ± 2.83	30.8	18.0
TD	Thoracic depth (cm)	9.71 ± 1.38	14.0	7.00
CL	Carcass length (cm)	31.7 ± 3.18	37.5	22.2
TL	Thigh length (cm)	5.27 ± 0.97	7.00	3.40
LC	Lumbar circumference (cm)	13.9 ± 2.02	18.9	9.40
CCI	Carcass compactness index (g/cm)	21.6 ± 5.15	37.0	11.5
TCM	Total carcass muscle (g)	488 ± 171	1045	191
TCB	Total carcass bone (g)	207 ± 61.4	344	80.2

SD = Standard deviation.

**Table 2:** Correlation coefficients (r) between body weight, carcass traits, and post mortem measurements in growing rabbits.

	HCW	CCW	DL	TD	CL	TL	LC	CCI	TCM	TCB
BW	0.97 *	0.98 *	0.70 *	0.86 *	0.81	0.56	0.87 *	0.96 *	0.97 *	0.68 *
HCW		0.99 *	0.74 *	0.83 *	0.84	0.50 *	0.91 *	0.97 *	0.98 *	0.73 *
CCW			0.76 *	0.84 *	0.83 *	0.52 *	0.90 *	0.97 *	0.97 *	0.76 *
DL				0.54 *	0.95 *	0.19 ns	0.60 *	0.61 *	0.67 *	0.77 *
TD					0.67 *	0.62 *	0.81 *	0.82 *	0.86 *	0.47 *
CL						0.45 *	0.69 *	0.71 *	0.78 *	0.75 *
TL							0.50 *	0.51 **	0.55 *	0.16 ns
LC								0.91	0.90 *	0.62 *
CCI									0.96 *	0.68 *
TCM										0.59 *

Correlations followed by superscript indicate * *p* < 0.001; ** *p* < 0.05; ns—non-significant. BW—body weight (g); HCW—hot carcass weight (g); CCW—cold carcass weight (g); DL—dorsal length (cm), TD—thoracic depth (cm), CL—carcass length (cm), TL—thigh length (cm), LC—lumbar circumference (cm), CCI—carcass compactness index (g/cm), TCM—total carcass muscle (g); TCB—total carcass bone (g).

**Table 3:** Regression equations to predict body weight and carcass traits using post mortem measurements in growing rabbits.

No	Equations	R^2^	MSE	RMSE	AIC	BIC	CV	*p*-Value
1	TCM (g) = −214 (±28.4 ***) + 32.3 (±1.27 ***) × CCI	0.93	2058.5	45.4	375.8	376.1	9.30	<0.0001
2	TCM (g) = −455 (±60.2 ***) + 10.8 (±2.47 ***) × CL + 27.5 (±1.52 ***) × CCI	0.95	1487.4	38.6	360.8	362.9	7.91	<0.0001
3	TCB (g) = −236 (±53.1 ***) + 16.7 (±1.98 ***) × DL	0.60	1543.2	39.3	361.6	362.9	18.9	<0.0001
4	TCB (g) = −205 (±49.3 ***) + 12.1 (±2.29 ***) × DL + 4.13 (±1.27 *) × CCI	0.67	1282.7	35.8	353.5	355.7	17.3	<0.0001

Values within parentheses are the standard errors (SEs) of the parameter estimates. BW—body weight (g); HCW—hot carcass weight (g); CCW—cold carcass weight (g); DL—dorsal length (cm), TD—thoracic depth (cm), CL—carcass length (cm), TL—thigh length (cm), LC—lumbar circumference (cm), CCI—carcass compactness index (g/cm), TCM—total carcass muscle (g); TCB—total carcass bone (g); AIC—Akaike information criterion; MSE—mean square error, RMSE—Root of MSE, BIC—Bayesian information criterion; CV—coefficient of variation (%), R^2^—coefficient of determination. Values in parentheses are the standard errors (SEs) of the parameter estimates. *: *p* < 0.05; ***: *p* < 0.001.

**Table 4:** Internal *k*-fold cross-validation of the proposed models.

Equation No.	*n*	R^2^	MSEP	RMSEP	MAE
2	50	0.93	1112.9	33.4	29.8
4	50	0.57	1336.6	36.6	28.2

MSEP—Mean Square Error of Prediction, RMSEP—root mean square error of prediction, R^2^—coefficient of determination; MAE—mean absolute error

## Data Availability

The data presented in this study are available on request from the corresponding author.
